# Fibroblast Growth Factor (FGF) 23 Regulates the Plasma Levels of Parathyroid Hormone In Vivo Through the FGF Receptor in Normocalcemia, But Not in Hypocalcemia

**DOI:** 10.1007/s00223-017-0333-9

**Published:** 2017-10-23

**Authors:** Maria L. Mace, Eva Gravesen, Anders Nordholm, Klaus Olgaard, Ewa Lewin

**Affiliations:** 1 Department of Nephrology, Herlev Hospital, University of Copenhagen, 2730 Copenhagen, Denmark; 2 Department of Nephrology, Rigshospitalet Copenhagen, University of Copenhagen, Copenhagen, Denmark

**Keywords:** PTH, FGF23, FGFR, PD173074, Hypocalcemia

## Abstract

The calcium and phosphate homeostasis is regulated by a complex interplay between parathyroid hormone (PTH), fibroblast growth factor 23 (FGF23), and calcitriol. Experimental studies have demonstrated an inhibitory effect of FG23 on PTH production and secretion; the physiological role of this regulation is however not well understood. Surprisingly, in uremia, concomitantly elevated FGF23 and PTH levels are observed. The parathyroid gland rapidly loses its responsiveness to extracellular calcium in vitro and a functional parathyroid cell line has currently not been established. Therefore, the aim of the present investigation was to study the impact of FGF23 on the Ca^2+^/PTH relationship in vivo under conditions of normocalcemia and hypocalcemia. Wistar rats were allocated to treatment with intravenous recombinant FGF23 and inhibition of the FGF receptor in the setting of normocalcemia and acute hypocalcemia. We demonstrated that FGF23 rapidly inhibited PTH secretion and that this effect was completely blocked by inhibition of the FGF receptor. Furthermore, inhibition of the FGF receptor by itself significantly increased PTH levels, indicating that FGF23 has a suppressive tonus on the parathyroid gland’s PTH secretion. In acute hypocalcemia, there was no effect of either recombinant FGF23 or FGF receptor inhibition on the physiological response to the low ionized calcium levels. In conclusion, FGF23 has an inhibitory tonus on PTH secretion in normocalcemia and signals through the FGF receptor. In acute hypocalcemia, when increased PTH secretion is needed to restore the calcium homeostasis, this inhibitory effect of FGF23 is abolished.

## Introduction

The primary role of parathyroid hormone (PTH) is to prevent and reverse acute hypocalcemia by mobilizing calcium from bone, stimulating renal Ca^2+^ reabsorption, and by promoting the production of the active vitamin D metabolite calcitriol [1]. Extracellular Ca^2+^ is the main determinant of the secretion of PTH from the parathyroid glands. The parathyroid glands express abundant calcium-sensing receptor (CaSR) mRNA and protein [2]. Small changes in extracellular Ca^2+^ trigger CaSR-mediated intracellular Ca^2+^ signaling and PTH secretion [3].

The Ca^2+^/PTH relationship is described by a steep sigmoidal curve. A small decline in Ca^2+^ below normal will result in a dramatic increase in the PTH secretion. The maximal secretory rate, the upper part of the curve, is achieved after just a slight degree of hypocalcemia. Induction of even mild hypercalcemia will, in contrast, result in a modest decline of PTH secretion to its minimal level [1].

Several factors are involved in the complexity of the Ca^2+^/PTH relationship such as intraglandular degradation of PTH, recruitment of parathyroid cells in an active state, autocrine/paracrine factors, hysteresis, rate-dependent control of the PTH response to reduction of Ca^2+^, and several intraglandular factors. The detailed mechanism behind the complexity of the Ca^2+^ regulated PTH secretion is still not well understood [4, 5]. The newly described crystal structure of CaSR extracellular domain brought important and fascinating new information on the mechanisms by which Ca^2+^ and other ions control the parathyroid function [6, 7]. Crystal structures of the resting and active conformation reveal multiple binding sites for Ca^2+^ and PO_4_^3−^ ions. While Ca^2+^ stabilizes the active state, PO_4_^3−^ ions reinforce the inactive conformation of CaSR [7]. This finding further emphasizes that calcium and phosphate homeostases are interrelated at the level of parathyroids as previously indicated by our group and others showing direct regulatory effect of phosphate on PTH secretion [8–10].

Fibroblast growth factor 23 (FGF23) activates the FGF receptor (FGFR) in the presence of the obligate co-receptor αKlotho and the parathyroid gland expresses both FGFRs and αKlotho [11–13]. FGF23 has been proposed to have several effects on the parathyroid glands such as decreasing PTH gene expression and secretion, increasing the parathyroid glands expressions of CaSR, vitamin D receptor (VDR), and αKlotho, altogether contributing to the suppression of PTH [12, 14]. Recently, it was shown that FGF23 via activation of the αKlotho/FGF receptor signaling is positively associated with parathyroid cell proliferation [15].

Clinical observations of either high or low FGF23 levels have shown very varied plasma PTH levels [16]. As alteration in FGF23 levels also induces changes in the levels of calcitriol, Ca^2+^, and phosphate, it can be difficult to unravel the direct effect of FGF23 on PTH levels. Experimental studies on the direct effect of FGF23 on the parathyroid gland have shown an inhibitory effect on PTH mRNA and hormone secretion [12, 14]. However, the physiological function of the inhibitory effect of FGF23 on the parathyroid gland’s PTH gene expression and PTH secretion is not well understood. The parathyroid gland loses rapidly its responsiveness to extracellular calcium ex vivo and there has currently not been established a functional parathyroid cell line [17, 18]. Therefore, the aim of the present investigation was to study the impact of FGF23 on the Ca^2+^/PTH relationship in vivo under conditions of normocalcemia and hypocalcemia. The present study demonstrates for the first time that FGF23 has an inhibitory tonus on PTH secretion when plasma Ca^2+^ is within normal range. At low plasma Ca^2+^, when increased PTH secretion is needed to restore Ca^2+^ levels, this inhibitory effect of FGF23 on PTH secretion is abolished.

## Materials and Methods

### Animals

Adult male Wistar rats (200 g) were used for the study (Charles Rivers Laboratories, GmbH, Germany). They were housed in an accredited facility with a 12-h light/dark circle. The rats had free access to water and standard diet containing 0.7% calcium, 0.5% phosphorus, and 600 IU vitamin D per kg (Altromin, Lage, Germany).

### Study Approval

The experiments were conducted in accordance to the national guidelines for care and use of laboratory animals. The experimental protocols were approved by the Danish Animal Experiments Inspectorate (license 2012-DY-2934-00023).

### Experimental Protocols

The rats were allocated to one of the following models:

1. Treatment with recombinant FGF23 (rFGF23) in normocalcemic condition (*n* = 4).
2. Treatment with FGF Receptor inhibitor (FGFRi) and rFGF23 in normocalcemic condition (*n* = 9).
3. Treatment with a combination of rFGF23 or vehicle and induction of acute hypocalcemia (*n* = 12).
4. Treatment with a combination of FGFRi or vehicle and induction of acute hypocalcemia (*n* = 12).

The rats were anesthetized with pentobarbital (50 μg/kg) intraperitoneally. A catheter was placed in the femoral vein for intravenous infusion and a catheter was placed in the femoral artery for blood sampling. Blood was drawn at baseline and at times 0, 5, 10 min, and subsequently every 10 min. Same volume of isotonic saline was administered after blood sampling to restore blood volume.

We used full-length human rFGF23 (R&D Systems, Minneapolis, MN) dissolved in sterile phosphate-buffered saline according to manufacturer’s recommendation and diluted in isotonic saline to a volume of 100 μl. The rFGF23 was given as an intravenous bolus injection. Vehicle consisted of 100 μl isotonic saline. The FGF receptor inhibitor PD173074 (L&C Laboratory, Woburn, MA) 40 mg was dissolved in 200 μl 96% ethanol and administered by oral gavage. Vehicle consisted of 200 μl 96% ethanol. In model 2, increasing doses of rFGF23 were administered to groups of rats, 0.1 μg rFGF23 (*n* = 5), 1 μg rFGF23 (*n* = 2), and 10 μg rFGF23 (*n* = 2). Acute hypocalcemia was induced by a continuous intravenous infusion of 40 mM EGTA (ethylene-bis(oxyethylenenitrilo)tetraacetic acid; Sigma, USA) at a rate of 3.0 ml/h [19].

### Biochemistry

Plasma Ca^2+^ was measured by a calcium selective electrode (ABL505, Radiometer, Copenhagen, Denmark). Plasma FGF23 was measured using the intact FGF23 enzyme-linked immunosorbent assay (Kainos Laboratory, Tokyo, Japan) measuring both rat and human FGF23. The intra- and inter-assay coefficients of variation in our laboratory were 2.5 and 5%, respectively [13]. PTH was measured by an ELISA method (Immutopics, San Clemente, CA). The inter-assay and intra-assay coefficients of variation of this PTH ELISA were, respectively, 9 and 4% in our laboratory [5].

### Statistical Analyses

Normal distribution of data was assessed in GraphPad Prism 7. Data are expressed as mean ± SEM. Statistical significance was tested using paired or unpaired two-sided *t* test calculated in Excel and GraphPad Prism 7. Significance level was set at *p* ≤ 0.05.

## Results

### Effect of FGF23 and FGF Receptor Inhibition on the Plasma Levels of PTH in Normocalcemia

**Fig. 1:**
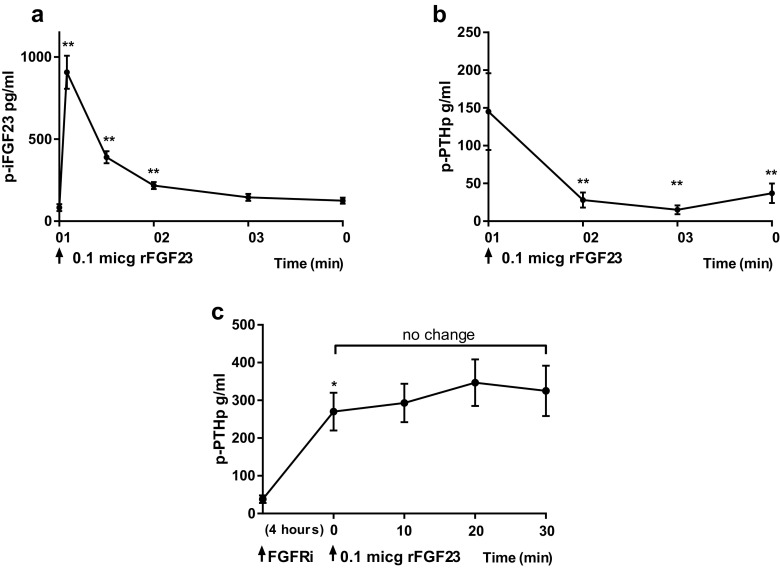
FGF23 regulates the plasma levels of PTH. **a** rFGF23 was administered intravenously to normal rats and resulted in a large increase in plasma intact FGF23. **b** The rFGF23 resulted in a significant decrease in plasma PTH levels after 10 min and the following PTH levels remained low. **c** Administration of the FGF receptor inhibitor (FGFRi), PD173074, resulted in a significant increase in PTH levels after 4 h. When rFGF23 was given after prior FGFRi, there was no effect of FGF23 on PTH levels. Data are expressed as mean ± SEM. *p < 0.05, **p < 0.01, *n* = 4 in (**a** and **b**), *n* = 5 in (**c**)

To study the effect of FGF23 on PTH levels in plasma, we administered rFGF23 by an intravenous bolus injection in normal rats. The rats were given 0.1 µg rFGF23 resulting in a significant increase in plasma levels of intact FGF23 from 83 ± 21 to 907 ± 101 pg/ml (*p* < 0.01). The rFGF23 was rapidly cleared from the circulation and FGF23 levels became close to baseline levels after 20–30 min (Fig. 1a). This is in accordance with our previous finding of a short half-life of FGF23 [20]. The rFGF23 caused a significant decrease in the plasma levels of PTH already at 10 min (*p* < 0.01) and subsequent lower levels of PTH (Fig. 1b).

In order to examine whether FGF23 was signaling through the FGF receptor, we combined administration of FGFRi PD173074 and rFGF23. FGFRi resulted in a significant decrease in plasma iFGF23 levels from 116 ± 37 to 21 ± 11 pg/ml (*p* < 0.05) and in a simultaneous increase in plasma levels of PTH from 38 ± 10 to 270 ± 50 pg/ml after 4 h (*p* < 0.05, Fig. 1c), indicating that FGF23 signaling via the FGFR has an inhibitory tonus on PTH secretion. When 0.1 µg of rFGF23 was given after prior FGFRi, it had no suppressive effect on the plasma levels of PTH (Fig. 1c). Increasing doses, 1 and 10 µg, of rFGF23 resulted in very high plasma levels of FGF23 around 12,000 and 120,000 pg/ml, respectively. However, these high FGF23 levels did not suppress PTH levels when the FGFR was inhibited (data not shown).

### Effect of FGF23 on the Plasma Levels of PTH in During Hypocalcemia

**Fig. 2:**
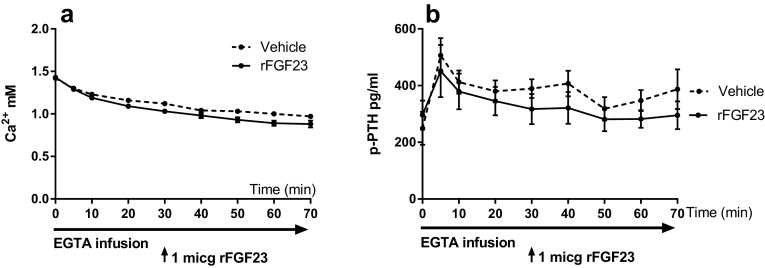
FGF23’s suppressive effect on PTH secretion is abolished at low plasma Ca^2+^ levels. **a** Plasma Ca^2+^ was rapidly decreased by an EGTA infusion (*p* < 0.001). After 30 min, rFGF23 was administered intravenously. **b** The acute lowering of plasma Ca^2+^ resulted in a dramatic increase in PTH and a higher plateau of maximal secretion (*p* < 0.05). In hypocalcemia, the rFGF23 had no suppressive effect on the high PTH levels. Data are expressed as mean ± SEM, *n* = 6 in each group

To study the effect of FGF23 on PTH secretion in the setting of hypocalcemia, the parathyroid glands’ PTH secretion was stimulated by induction of acute hypocalcemia using a continuous intravenous EGTA infusion. The EGTA infusion resulted in a significant drop in plasma Ca^2+^ levels (Fig. 2a) and after 5 min of infusion the maximum PTH secretory response was obtained (Fig. 2b). After 30 min of EGTA infusion and during maximal PTH secretion, an intravenous bolus of 1 µg of rFGF23 or vehicle was given. The rFGF23 injection significantly increased plasma levels of FGF23 to 1774 ± 400 versus 262 ± 51 pg/ml in the vehicle group (*p* < 0.01). In spite of these high FGF23 levels, rFGF23 had no suppressive effect on PTH levels during hypocalcemia (Fig. 2b).

### Effect of FGFRi on the Plasma Levels of PTH in During Hypocalcemia

**Fig. 3:**
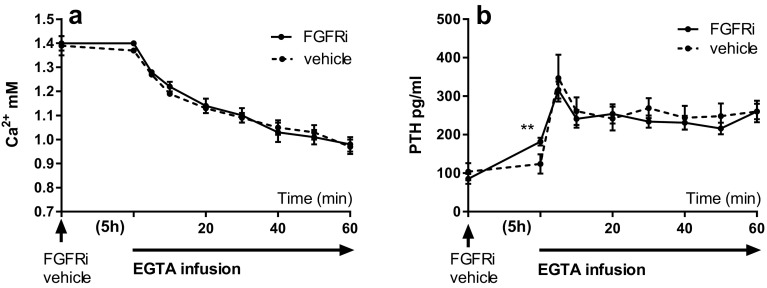
FGF receptor inhibition has no effect on PTH secretion in hypocalcemia. **a** FGFRi or vehicle was administered prior to induction of acute hypocalcemia by a continuous EGTA infusion that rapidly decreased plasma Ca^2+^ (*p* < 0.001). **b** FGFRi resulted again in a significant increase in PTH levels (*p* < 0.01). The rapid increase in PTH levels in response to acute hypocalcemia was however similar between the two groups. Data are expressed as mean ± SEM. **p < 0.01 *n* = 6 in each group

In order to study the effect of FGFRi on PTH secretion in the setting of hypocalcemia, we administered the FGFRi PD173074 or vehicle prior to induction of acute hypocalcemia by EGTA infusion. The FGFRi treatment alone had no effect on plasma levels of Ca^2+^ (Fig. 3a). The EGTA infusion rapidly decreased the plasma levels of Ca^2+^ in rats treated with vehicle and in rats treated with FGFRi with no difference between the two groups (Fig. 3a). Again FGFRi resulted in a significant increase in plasma PTH levels from 85 ± 13 to 182 ± 10 pg/ml (*p* < 0.01). Stable PTH levels were found in the vehicle-treated rats, 104 ± 22 versus 124 ± 25 pg/ml (Fig. 3b). The parathyroid glands responded immediately to the decrease in plasma Ca^2+^ with a large and significant increase in plasma PTH levels, obtaining maximum levels already after 5 min of EGTA infusion. In the vehicle-treated rats PTH increased from 124 ± 25 to 347 ± 61 pg/ml (*p* < 0.01) and in the FGFRi-treated rats PTH increased from 184 ± 10 to 316 ± 22 pg/ml (*p* < 0.01). There was no difference in the maximum PTH secretory response to hypocalcemia in the two groups (Fig. 3b). The plasma Ca^2+^ concentration continued to decrease at a similar rate during the EGTA infusion in both groups and reached low levels of 0.97 ± 0.03 mM (vehicle rats) and 0.98 ± 0.03 mM (FGFRi rats) after 60 min. The maximal plateau of PTH secretion obtained after the transient maximum PTH secretory response was the same in the two groups (Fig. 3b).

## Discussion

We used an in vivo model to examine the regulatory function of FGF23 on the plasma levels of PTH and demonstrated that FGF23 has an inhibitory tonus on PTH secretion, but only when the plasma concentration of Ca^2+^ is within normal range. At low plasma Ca^2+^ levels, when increased PTH secretion is needed in order to restore normal Ca^2+^ levels, this inhibitory effect of FGF23 is abolished. Furthermore, we clearly demonstrated that the suppressive effect of FGF23 in normocalcemia is mediated through the FGF receptor.

A suppressive effect of FGF23 on PTH production and secretion has previously been shown by Ben-Dov et al. They demonstrated a rapid suppressive effect of intravenous FGF23 on plasma PTH levels. This was mediated by phosphorylation of ERK1/2 as an indication of activation of the MAPK pathway [12]. Furthermore, they injected rats with FGF23 daily for 5 days and confirmed the decrease in plasma PTH levels by a decrease in PTH mRNA. Their studies were done in normocalcemia [12]. A suppressive effect of daily FGF23 injections on PTH levels in normal rats and activation of the MAPK/ERK1/2 signaling pathway has also been shown by Canalejo et al. [21]. Activation of the αKlotho-FGFR complex by FGF23 has been shown to conduct downstream signaling through the MAPK/ERK1/2 pathway and this pathway is activated by FGF23 in the kidney [22]. Krajisnik et al. found ex vivo in bovine parathyroid glands a dose-dependent increase in Egr-1 mRNA, an early response gene of the MAPK/ERK1/2 signaling pathway, after co-incubation with two doses of recombinant FGF23 for 1 h and later a decrease in PTH mRNA and PTH concentration after 12 and 24 h, respectively [14]. Even though they found an early activation of the MAPK/ERK1/2 pathway, the effect of FGF23 on PTH levels was very late as compared to our in vivo results and to the study of Ben-Dov et al. The new findings in the present investigation provide a better understanding on how and when this inhibitory effect of FGF23 on PTH secretion is mediated and stress the impact of normocalcemia in physiological and pathophysiological conditions for the regulation to prevail. We confirm that the effect of FGF23 in normocalcemia is mediated via the FGF receptor in the parathyroid gland as shown in the present investigation by the use of a pharmacological FGF receptor inhibitor.

FGF23 is known to activate FGFR1 isoform IIIc, FGFR3 isoform IIIc, and FGFR4 [11]. Binding of the FGF ligand causes a dimerization of the FGF receptor and induces autophosphorylation of the intracellular domain and activation of downstream signaling of MAPK/ERK1/2, PI3 K/AKT, STAT, and PLCγ pathways [23]. We and others have previously shown that the parathyroid gland expresses high levels of FGFR1, FGFR2 and to a less extent FGFR3 and FGFR4 [13, 15]. We used a pan FGFR inhibitor in the present study and are therefore not able to identify which subtype of the FGF receptors that FGF23 activates in the parathyroid gland. Furthermore, PD173074 also inhibits the tyrosine kinase activity of vascular endothelial growth factor (VEGF) receptor. As such, an effect of this compound on other receptors than FGFR in the parathyroids cannot be excluded.

Upon stimulation of CaSR a number of intracellular signal transduction pathways are activated, among them are phosphoinositide-specific phospholipase C and MAPK/ERK1/2. Characteristic for PTH secretion is that it is increased at low intracellular Ca^2+^ levels and suppressed by an increase in intracellular Ca^2+^ level, where the latter is caused by activation of CaSR [3]. Activation of the FGFRs leads to phosphorylation of PLCγ, which hydrolyses phosphatidylinositol 4,5 biphosphate to produce inositol triphosphate (IP_3_). IP_3_ increases intracellular Ca^2+^ concentration [23]. FGF23 has been demonstrated to increase the intracellular Ca^2+^ level in cardiomyocytes [24]. Potentially, this mechanism might explain the rapid inhibitory effect of FGF23 on PTH secretion. Further research is however needed to clarify the signal transduction of FGF23 on intracellular calcium in the parathyroid cell.

Even though most studies confirm the obligatory function of αKlotho in converting the FGFR to be specific for FGF23 signaling, a recent study questions the necessary co-receptor function of αKlotho in the parathyroid gland, as a specific deletion of the αKlotho gene in the parathyroid gland did not abolish the effect of FGF23 [25]. In wildtype mice, FGF23 activated the MAPK/ERK1/2 pathway in the parathyroid glands and suppressed PTH levels. However, in genetically manipulated mice with a specific αKlotho gene deletion in the parathyroid glands, FGF23 activated the calcineurin pathway and suppressed PTH levels [25].

Kawakami et al. used genetic-manipulated models, specific deletion of FGFRs and αKlotho in the parathyroid glands of mice, and found in wildtype mice an acute suppressive effect of FGF23 on PTH secretion after 1 h in accordance to our findings [15]. In the FGFR1-3 and αKlotho knocked-out models, the effect of FGF23 on PTH levels was abolished further supporting our findings of FGF23 signaling through the FGF receptor in the parathyroid gland.

Chronic kidney disease causes disturbances in the mineral balance and concomitant high levels of plasma FGF23 and PTH. In a previous investigation, we administered the FGF receptor inhibitor, PD173074, to uremic normocalcemic rats and found a large increase in plasma PTH indicating that the suppressive tonus of FGF23 on the parathyroid gland is still present in uremia, when plasma Ca^2+^ is within normal range [26]. Galitzer et al. also found a suppressive effect of FGF23 on the parathyroid glands in uremic rats after 2 weeks of uremia induced by adenine diet [27]. These uremic rats had the same expression of αKlotho in the parathyroid gland as normal rats, but reduced FGFR1 expression. After 6 weeks of uremia, both αKlotho and FGFR1 expressions were reduced and they found no effect of FGF23 on the parathyroid gland [27]. However, other studies suggest that the parathyroid glands in uremia might be resistant to the high plasma levels of FGF23 as shown both in vivo and in vitro by Canalejo et al [21]. It is currently believed that parathyroid resistance to FGF23 could be due to downregulation of αKlotho in the parathyroid gland [16, 27, 28]. However, we did not find a downregulation of parathyroid αKlotho expression in a previous study on uremic 5/6-nephrectomized rats and other researchers have reported a varied αKlotho expression within a uremic parathyroid gland [13, 29].

The present investigation is dealing with the acute minute-to-minute regulation of the Ca^2+^/PTH relationship. Hypocalcemia is a common complication to kidney failure and therefore on the basis of our present results we propose an additional mechanism behind the resistance of the parathyroid gland to FGF23 in uremia, as the low plasma Ca^2+^ levels overrule the inhibitory effect of FGF23. Further studies are needed to examine the resistance in chronic hypocalcemia, another frequent finding in chronic uremia. Collectively all studies may illustrate the gradual change from normal physiology to a severe pathophysiology with development of a more and more dysplastic autonomous secreting parathyroid gland with downregulation of CaSR, VDR, αKlotho, and FGFR1.

Our results in vivo are in contrast to an ex vivo study on rat parathyroid glands, where FGF23 still had a suppressive effect on PTH secretion and PTH mRNA when the parathyroid cells were incubated at low calcium for 6 h [21]. These discrepant results may be due to in vivo versus in vitro models. Abolishment of an inhibitory effect during hypocalcemia is known from other endocrine regulatory mechanisms as it has been demonstrated for calcitriol’s regulation of PTH transcription [30]. Thus, in chronic hypocalcemia there is a marked increase in calcitriol, which has no suppressive effect on PTH levels; they are significantly increased as well [30]. The mechanism behind these findings has been proposed to be due to the binding of calreticulin to VDR-RXRβ to VDRE of the PTH promoter [31]. The levels of Ca^2+^ have been reported also to control FGF23 levels [32, 33]. Therefore, the complex interplay between the calcium and phosphate regulating hormones may also be influenced by the extracellular Ca^2+^ concentration and thus facilitate the ability of the organism to maintain the crucial Ca^2+^ homeostasis for normal cellular and organ function [20, 34].

All FGF receptors have a cluster of acidic amino acids in their extracellular region. It has been demonstrated that FGFR1 binds Ca^2+^, although low Ca^2+^ in the media did not alter the binding of the ligand FGF2 in a cell line [35]. It is however not known if this region affects the interaction in ligand-receptor binding for other FGFRs and ligands. It has been proposed that the binding of a divalent cation will result in a loop configuration in this region that could alter the 3D configuration of the receptor and facilitate ligand binding. Thus a study on cloned FGF receptors found a weaker ligand-receptor binding when rat parathyroid cells were incubated at low calcium concentration [36].

FGF23’s suppressive effect on PTH may be part of a classical endocrine feedback loop, where PTH stimulates FGF23 expression via the PTH receptor 1 and possibly by activation of Nurr1 transcription [37–40]. Both hormones have phosphaturic effects, yet they impose a counter regulation on calcitriol with a stimulatory effect of PTH and an inhibitory effect of FGF23. Furthermore, the regulatory loop between FGF23 and PTH seems to be complex and also depending on the extracellular Ca^2+^ concentration, as shown in the present data. Our study is dealing with the acute effect of FGF23 on PTH secretion, and it has to be emphasized that most clinical scenarios of high FGF23 are chronic and the interpretation from the present acute experiments cannot directly be transferred to image the situation in long-term increase of FGF23 levels.

In conclusion, FGF23 has an inhibitory tonus on PTH secretion when extracellular Ca^2+^ calcium is within normal range. This regulation is part of a complex endocrine system consisting of FGF23, PTH, and calcitriol, which maintains Ca^2+^ and phosphate homeostasis. In hypocalcemia, when increased PTH secretion is needed to restore the calcium homeostasis, this inhibitory effect of FGF23 is abolished.
